# Investigating the cut-off values of captopril challenge test for primary aldosteronism using the novel chemiluminescent enzyme immunoassay method: a retrospective cohort study

**DOI:** 10.1038/s41440-024-01594-x

**Published:** 2024-03-08

**Authors:** Yuta Tezuka, Kei Omata, Yoshikiyo Ono, Kengo Kambara, Hiroki Kamada, Sota Oguro, Yuto Yamazaki, Celso E. Gomez-Sanchez, Akihiro Ito, Hironobu Sasano, Kei Takase, Tetsuhiro Tanaka, Hideki Katagiri, Fumitoshi Satoh

**Affiliations:** 1 Department of Diabetes, Metabolism, and Endocrinology, Tohoku University Hospital, 1-1 Seiryo-machi, Aoba-ku, Sendai, Miyagi 980-8574 Japan; 2 Division of Nephrology, Rheumatology, and Endocrinology, Tohoku University Graduate School of Medicine, 2-1 Seiryo-machi, Aoba-ku, Sendai, Miyagi 980-8575 Japan; 3 Medical Systems Research & Development Center, Medical Systems Business Division, FUJIFILM Corporation, 6-1, Takata-cho, Amagasaki-shi, Hyogo 661-0963 Japan; 4 Department of Diagnostic Radiology, Tohoku University Graduate School of Medicine, 2-1 Seiryo-machi, Aoba-ku, Sendai, Miyagi 980-8575 Japan; 5 Department of Pathology, Tohoku University Graduate School of Medicine, 2-1 Seiryo-machi, Aoba-ku, Sendai, Miyagi 980-8575 Japan; 6 Department of Pharmacology and Toxicology, University of Mississippi Medical Center, 2500 North State Street, Jackson, MS 39216 USA; 7 Department of Urology, Tohoku University Graduate School of Medicine, 1-1 Seiryo-machi, Aoba-ku, Sendai, Miyagi 980-8574 Japan

**Keywords:** Primary aldosteronism, Captopril challenge test, Chemiluminescent enzyme immunoassay, Confirmatory test

## Abstract

The measurement evolution enabled more accurate evaluation of aldosterone production in hypertensive patients. However, the cut-off values for novel assays have been not sufficiently validated. The present study was undertaken to validate the novel chemiluminescent enzyme immunoassay for aldosterone in conjunction with other methods. Moreover, we also aimed to establish a new cut-off value for primary aldosteronism in the captopril challenge test using the novel assay. First, we collected 390 plasma samples, in which aldosterone levels measured using liquid chromatography-mass spectrometry ranged between 0.18 and 1346 ng/dL. The novel chemiluminescent enzyme immunoassay showed identical correlation of plasma aldosterone with liquid chromatography-mass spectrometry, in contrast to conventional radioimmunoassay. Further, we enrolled 299 and 39 patients with primary aldosteronism and essential hypertension, respectively. Plasma aldosterone concentrations measured using the novel assay were lower than those measured by radioimmunoassay, which resulted in decreased aldosterone-to-renin ratios. Subsequently, positive results of the captopril challenge test based on radioimmunoassay turned into “negative” based on the novel assay in 45% patients with primary aldosteronism, using the conventional cut-off value (aldosterone-to-renin activity ratio > 20 ng/dL per ng/mL/h). Receiver operating characteristic curve analysis demonstrated that aldosterone-to-renin activity ratios > 8.2 ng/dL per ng/mL/h in the novel assay was compatible with the conventional diagnosis (sensitivity, 0.874; specificity, 0.980). Our study indicates the great measurement accuracy of the novel chemiluminescent enzyme immunoassay for aldosterone, and the importance of measurement-adjusted cut-offs in the diagnosis of primary aldosteronism.

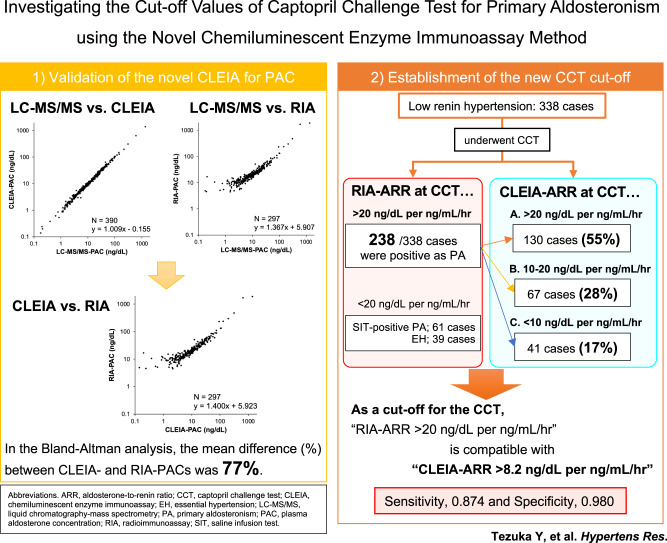

## Introduction

Primary aldosteronism (PA), the most common cause of endocrine hypertension, affects an estimated 5–10% of all patients with hypertension [1–3]. The prevalence of PA increases by approximately 20% among patients with resistant hypertension, 10% in those with severe hypertension, and 6% among those with otherwise uncomplicated hypertension. However, only a small fraction of patients undergo screening for PA and receive adequate treatment [4–6]. In PA, renin-independent aldosterone overproduction aberrantly activates mineralocorticoid signaling, contributing to elevated blood pressure and subsequent renal and cardiovascular risks [7]. Several meta-analyses have clearly indicated the remarkably increased risks of coronary artery diseases, atrial fibrillation, stroke, and chronic kidney disease in patients with PA compared to those with essential hypertension (EH), which could be mitigated by mineralocorticoid receptor blockers (MRBs) or eliminated by adrenalectomy, depending on the PA subtypes [8–11]. Therefore, timely testing and treatment optimization are crucial to minimize the comorbidities associated with PA.

The current diagnostic strategy of PA consists of three major steps: screening, confirmatory tests, and laterality identification of hyperaldosteronism. The aldosterone-to-renin ratio (ARR) is the recommended screening test for all hypertensive patients with the possibility of PA [12]. Then, these patients generally undergo any of following confirmatory tests to establish the diagnosis of PA: captopril challenge test (CCT), saline infusion test (SIT), upright furosemide test, or oral salt loading test [12, 13]. In Japan, CCT is the most employed tool to discriminate PA cases from other cases with low renin. This is due to the easiness and safeness with which the procedure can be performed [13]. Captopril, an angiotensin-converting enzyme inhibitor, interrupts renin-angiotensin-aldosterone system (RAAS), which results in increase in renin and decrease in aldosterone. Patients with PA typically demonstrate persistent hyperaldosteronism with suppressed renin, even after captopril loading. The distinguishable responses to captopril challenge between PA and other hypertension cases enables the identification of PA based on CCT. Similarly, other confirmatory tests can also be used to corroborate the diagnosis of PA. Further, localization of PA can be done primarily by adrenal venous sampling (AVS).

Recently, novel chemiluminescent enzyme immunoassays (CLEIAs) have been developed for commercial measurement of plasma aldosterone concentrations (PACs). This method reportedly has high sensitivity and reproducibility [14]. These assays have been proven to have a very high measurement accuracy that is compatible with that of the gold standard method, liquid-chromatography mass-spectrometry (LC-MS/MS) [15, 16]. The regression coefficient between the CLEIA and LC-MS/MS methods reportedly ranges between 0.98 and 1.07 with a smaller intercept than 1 ng/dL [14–16], indicating that the novel CLEIA methods can be used as alternate standard for PACs. Applying the CLEIAs to everyday practice, the Japan Endocrine Society proposed new criteria for PA screening and confirmatory tests [13]. However, the validation of those PA-diagnostic cut-offs has not been sufficiently performed. No study has compared PACs using the CLEIA and conventional radioimmunoassay (RIA) methods in the same blood samples obtained from confirmatory tests. The transition of PAC measurement from RIA to CLEIA could interfere with the classification of low renin hypertension as per the current criteria [17]. Therefore, we undertook this study to investigate the cut-off values of CCT using the novel CLEIA method, and compare it with the values obtained using the conventional technique.

## Methods

### Ethical considerations

All procedures performed in this study were in accordance with the tenets of Declaration of Helsinki. The study protocol was approved by Tohoku University Hospital institutional review board (2018-1-056 and 2023-1-229). Informed consent was obtained from all the participants prior to the commencement of the study.

### Study design and sample collection

We collected residual blood samples from patients who were admitted to our endocrine unit between 2015 and 2021 along with research information disclosure. After centrifugation, the plasma samples were immediately frozen and stored at −20 °C for aldosterone measurement in the future. This study had two specific objectives: 1) further validation of the novel CLEIA method for PACs by comparing the values obtained with those obtained using LC-MS/MS and RIA methods using a large number of samples; 2) establishment of a new cut-off value for CCT based on the CLEIA method. For fulfilling the first objective, we used plasma samples regardless of the collecting condition and patients’ comorbidity, yet considering the distribution of PACs measured using the conventional RIA method. On the other hand, we consecutively recruited patients with PA and EH between 2017 and 2020, who had both CCT and SIT with written informed consents for the second objective. Sample collection for these patients during CCT was done after 30 min rest in a supine position in the morning.

### PAC measurement using the novel CLEIA method (validation)

For determination of PAC by the new CLEIA method, we used Accuraseed aldosterone S kit (FUJIFILM Wako Pure Chemical Corporation, Osaka, Japan) and employed the two-step sandwich CLEIA method. This reagent uses a highly specific anti-aldosterone monoclonal antibody (A2E11) and an anti-aldosterone immune complex monoclonal antibody. A detailed description of the assay protocol is as follows: 25 μL of plasma was mixed with 50 μL of reagent 1, immuno-reaction buffer, and anti-aldosterone monoclonal antibody (A2E11) immobilized onto the magnetic particles MAGRAPID®. This mixture was then incubated for 180 s at 37 °C. The bound and free fractions were separated after incubation. Next, 50 μL of reagent 2 containing peroxidase-conjugated anti aldosterone immune complex monoclonal antibody was added and incubated for 180 s at 37 °C. After the incubation, the bound and free fractions were separated again. Finally, 100 µL of the substrate solution and 100 µL of hydrogen peroxide solution were added, and the amount of light emitted per unit time was measured. The assay for PAC was calibrated using Human Serum NMIJ CRM 6402.

To further validate this new CLEIA, we also measured PACs using the LC-MS/MS method (ASKA Pharmaceutical Co., Ltd., Tokyo, Japan) and the competitive RIA method (The SPAC-S aldosterone kit; Fujirebio Co., Ltd., Tokyo, Japan) in the same samples as described previously [14, 16].

### Eligibility of patients with PA and EH

For fulfilment of the second objective, the baseline measurement of PACs and plasma renin activities (PRAs) was performed using the available RIA and enzyme immunoassay methods, respectively. The diagnosis of PA was confirmed according to the Japanese guideline for PA [18]. Briefly, PACs and PRAs were initially evaluated in patients with hypertension after withdrawal of antihypertensive agents that interfered with RAAS. If the patients demonstrated ARR values > 20 ng/dL per ng/mL/h, they subsequently underwent both confirmatory tests to establish the diagnosis of PA or EH. In CCT, blood samples were collected at baseline and 90 min after 50 mg captopril loading to measure PACs and PRAs. We employed a PA criterion of ARRs > 20 ng/dL per ng/mL/h after the captopril challenge. For SIT, we drew blood samples at two time points: at baseline and after 2 L saline infusion over 4 h. The PA criterion for SIT was set as PACs > 6 ng/dL. Patients who demonstrated either or both positive results of these tests were categorized as having PA, while those who had both negative results were considered as having EH. Other secondary hypertensive diseases, including pheochromocytoma, Cushing syndrome, and renal artery stenosis, were excluded in all the patients. Following PA confirmation, the patients underwent AVS for determination of the subtype, unilateral or bilateral PA (UPA or BPA) as previously reported [19].

The following clinical information were obtained after reviewing the medical records of the patients: age, sex, weight, body mass index, blood pressure, antihypertensive drugs, renal function, serum potassium, potassium replacement, PACs (RIA), PRAs, and results of confirmatory tests and AVS.

### Statistical analysis

SPSS (version 28.0, Armonk, NY: IBM Corp) was used for performing all statistical analyses in this study. We dealt with the PRA values under the lower measurement limit (< 0.2 ng/mL/h) as 0.2 ng/mL/h in the statistical section. Passing-Bablok regression and Bland-Altman analysis were used for measurement comparison between CLEIA and LC-MS/MS or RIA. In the second part, non-parametric clinical parameters were presented as median with interquartile range. Mann-Whitney U test was used to compare non-parametric variables between the two groups. Chi-square test was applied for categorical variables. In addition, we employed the Wilcoxon signed-rank test to evaluate the changes in PACs, PRAs, and ARRs during the CCT. Spearman’s rank correlation analysis was used to investigate the association between the size of aldosterone-producing adenomas (APAs) and CLEIA-ARRs. Assessment of the discriminatory capacity of CCT was performed by plotting the receiver operating characteristic (ROC) curve. The area under the ROC curve (AUC) was used for ROC comparing analysis. The optimal cut-off value for CCT was determined based on the Youden index. Statistical significance was set at *p* < 0.05.

## Results

### Comparison of PACs measured using the novel CLEIA and other methods

#### PAC measurement by CLEIA vs LC-MS/MS as the gold standard

**Fig. 1:**
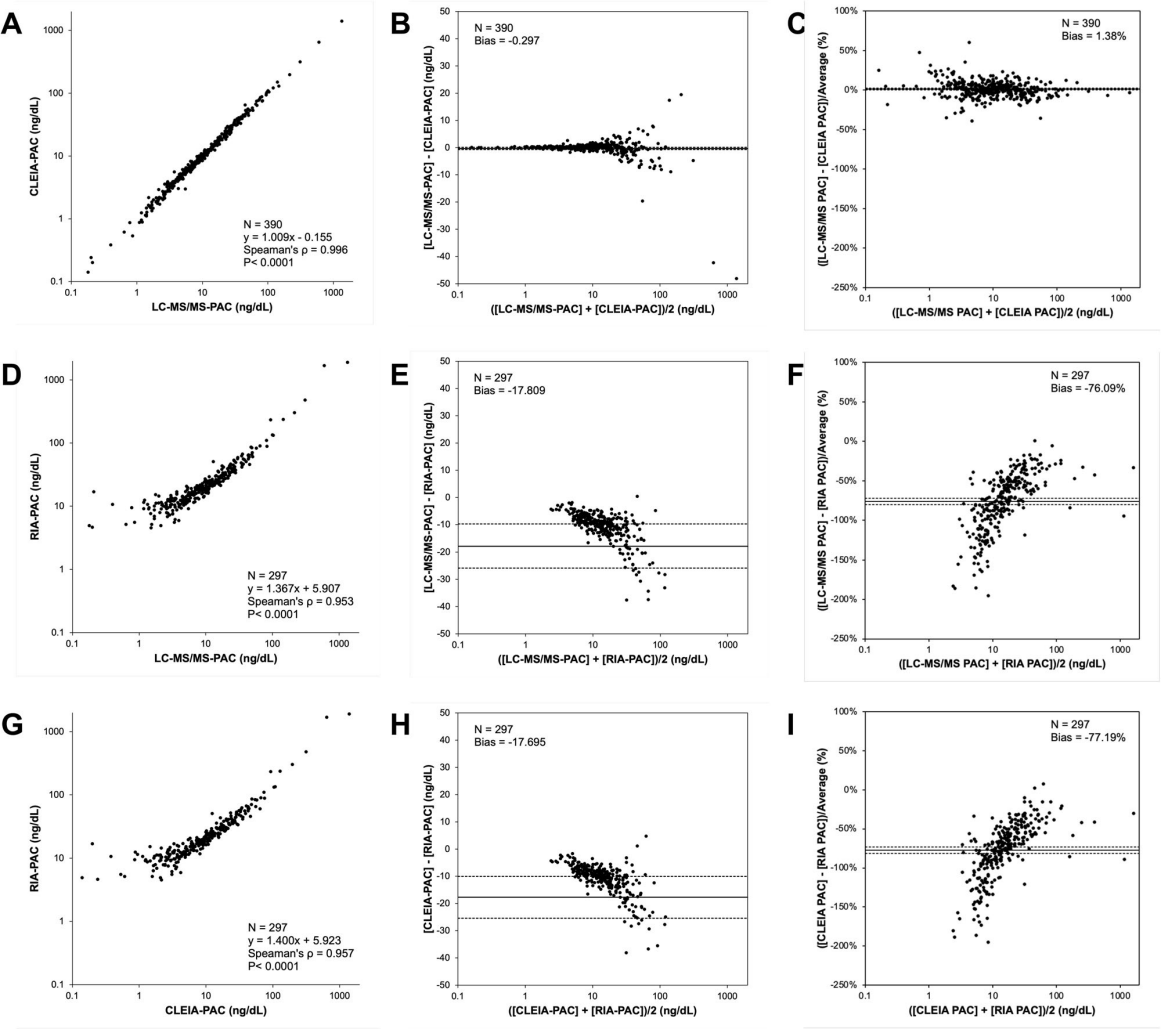
Validation of the novel CLEIA method for aldosterone measurement. Plasma aldosterone concentrations (PACs) measured using the novel chemiluminescent enzyme immunoassay (CLEIA) was validated with liquid chromatography-mass spectrometry (LC-MS/MS, the gold standard) and radioimmunoassay (RIA, the conventional assay). CLEIA vs. LC-MS/MS (**A**–**C**), RIA vs. LC-MS/MS (**D**–**F**) and CLEIA vs. RIA (**G**–**I**). Each validation was evaluated using the Passing-Bablok regression (**A**, **D**, **G**) and the Bland-Altman (**B**, **C**, **E**, **F**, **H**, **I**) analyses. In the Bland-Altman analysis, solid and broken lines indicate mean difference and its confidential interval, respectively

A total of 390 plasma samples including adrenal venous samples were available for PAC validation between CLEIA (CLEIA-PACs) and LC-MS/MS (LC-MS/MS-PACs). The LC-MS/MS-PACs widely ranged between 0.18 and 1346 ng/dL, with a median of 9.83 ng/dL. CLEIA-PACs correlated identically with the LC-MS/MS-PACs in the Passing- Bablok regression analysis, with the coefficient and intercept being 1.009 and −0.155, respectively (Fig. 1A). Bland–Altman analysis demonstrated a mean difference of −0.297 ng/dL with 95% confidence interval of −0.704 and 0.111 ng/dL between the two methods (Fig. 1B). The mean percentage of PAC difference between the two methods was 1.38% with 95% confidence interval of 0.33–2.44% (Fig. 1C).

#### PAC measurement using RIA, as a conventional method, and LC-MS/MS

Two hundred ninety-seven plasma samples which had formerly measured RIA-PACs were used for this validation. The distribution of LC-MS/MS-PAC was found to be similar; however, the median PAC was slightly lowered (9.30 ng/dL). Passing-Bablok regression analysis showed positive correlation between RIA-PACs and LC-MS/MS-PACs. However, the correlation was weak, particularly in the range under 10 ng/dL (Fig. 1D). RIA-PACs tended to be higher than LC-MS/MS-PACs, and the coefficient and intercept were 1.367 and 5.907, respectively. The mean differences with its 95% confidence interval in Bland–Altman analysis were as follows: −17.809 ng/dL (−25.870 to −9.748 ng/dL) and −76.09% (−80.20 to −71.98%) (Fig. 1E, F). The percentage of divergence between the two methods decreased with increase in PAC.

#### PAC measurement using the conventional RIA and the new standard CLEIA

Finally, we compared the CLEIA-based PACs with the RIA method using 297 plasma samples. The distributions of CLEIA- and RIA-PACs (median with interquartile range) were 9.30 [4.00, 17.70] and 19.30 [12.35, 30.50] ng/dL, respectively. Passing-Bablok regression analysis revealed that the correlation between CLEIA-PACs and RIA-PACs was similar to that between LC-MS/MS and RIA (Fig. 1D, G). The coefficient and intercept were 1.400 and 5.923, respectively. Bland–Altman analysis also showed similar mean differences (Fig. 1H, I). The mean percentage of PAC difference between the two groups was -77.19% with 95% confidence interval of −81.30 to −73.09%.

### Development of a new cut-off value for PA in CCT

#### Baseline characteristics of the study participants

A total of 338 patients with hypertension, 299 with PA, and 39 with EH were included in the study to fulfill the second objective (Table 1). Of the patients with PA, 196 had double positive results of CCT and SIT, and 103 had a positive result of either CCT or SIT (42 and 61, respectively). When compared with the EH group, the PA group showed similar characteristics in terms of age, body weight, blood pressure, serum potassium level, and renal function. However, the PA group comprised more men (*p* = 0.02) and required a higher amount of potassium replacement (*p* = 0.02). In the context of RAAS, the PA group showed higher RIA-PACs and RIA-ARRs along with lower PRAs in comparison to the EH group (*p* < 0.001). In the PA group, 277 (92.6%) patients underwent AVS after PA confirmation, which were identified as 109 (36.5%) UPAs and 168 (56.2%) BPAs. The remaining 22 (7.4%) patients with PA avoided AVS due to the mild nature of PA or personal reasons. The prevalence of UPA was found to be higher in those who had positive results of both confirmatory tests in comparison to others (52.0% vs. 6.8%; *p* < 0.01). This was in accordance with a previous report [20]. Based on the forementioned conversion formula for RIA to CLEIA, the CLEIA-PACs after 2 L saline infusion were estimated as 2.9 [0.6, 9.1] ng/dL in PA with positive RIA-CCT results.

**Table 1:** Baseline characteristics of the study participants

	PA				EH	*P* value
	All	Double positive	Positive only for CCT	Positive only for SIT		All PA vs. EH
	*N* = 299	*N* = 196	*N* = 42	*N* = 61	*N* = 39	
Male (n[%])	129 [43.1%]	94 [48.0%]	12 [28.6%]	23 [37.7%]	9 [23.1%]	0.02
Age (yr)	54 [46,64]	55 [47,64]	57 [53,66]	51 [42,57]	52 [47,64]	0.74
Body weight (kg)	64.6 [55.1, 74.8]	65.6 [55.4, 76.1]	59.6 [51.4, 64.9]	65.2 [55.9, 75.9]	61.0 [54.0, 73.6]	0.41
Body mass index (kg/m^2^)	24.4 [21.8, 27.0]	24.5 [22.0, 27.1]	23.0 [20.9, 24.9]	25.2 [22.2, 28.2]	25.1 [21.3, 28.0]	0.66
Systolic BP (mmHg)	137 [128, 148]	137 [128, 148]	135 [127, 143]	139 [130, 151]	141 [126, 150]	0.43
Diastolic BP (mmHg)	85 [77,91]	84 [77,90]	84 [77,91]	89 [81,95]	85 [78,95]	0.74
Anti-hypertensive drugs (n)	1 [1,2]	1 [1,2]	1 [1,2]	1 [1, 1]	1 [1,2]	0.76
eGFR (mL/min/1.73 m^2^)	78.0 [66.9, 91.0]	77.0 [66.0, 90.5]	80.5 [68.0, 91.0]	81.0 [68.5, 92.3]	77.0 [70.0, 91.8]	0.74
Serum potassium (mM)	3.8 [3.6, 4.0]	3.8 [3.5, 4.0]	4.0 [3.7, 4.2]	3.8 [3.7, 4.1]	3.9 [3.8, 4.1]	0.06
Propotion of Potassium replacement (*n*[%])	200 [66.9%]	150 [76.5%]	13 [31.0%]	37 [60.7%]	20 [51.3%]	0.05
Potassium dosage (mmol/day)	16.0 [0.0, 42.0]	24.0 [7.6, 54.0]	0.0 [0.0, 10.8]	10.8 [0.0, 26.0]	7.2 [0.0, 19.0]	0.02
PAC (ng/dL)	20.4 [15.6, 30.7]	22.7 [17.3, 36.1]	14.1 [12.1, 19.6]	18.6 [14.9, 22.6]	15.1 [12.1, 18.7]	<0.001
PRA (ng/mL/h)	0.20 [0.20, 0.40]	0.20 [0.20, 0.30]	0.20 [0.20, 0.30]	0.60 [0.40, 0.70]	0.50 [0.40, 0.68]	<0.001
ARR (ng/dL per ng/mL/h)	77.5 [45.1, 113.3]	95.8 [69.8, 151.0]	62.0 [45.5, 87.5]	33.8 [24.9, 44.3]	26.3 [22.2, 45.8]	<0.001
Number of PA subtypes (unilateral, bilateral, not specified)	109, 168, 22	102, 88, 6	2, 35, 5	5, 45, 11	-	

*PA* Primary aldosteronism, *EH* Essential hypertension, *CCT* Captopril challenge test, *SIT* Saline infusion test, *BP* Blood pressure, *eGFR* estimated glomerular function ratio, *PAC* Plasma aldosterone concentration (measured by the radioimmunoassay), *PRA* Plasma renin activity, *ARR* Aldosterone-to-renin ratio

Categorical and continuous variables are shown as number with percentages and median with interquartile range, respectively

Comparison of the variables between PA and EH was performed using Mann-Whitney U test or Chi-square test based on the variable category

#### Impact of CLEIA method on CCT

**Fig. 2:**
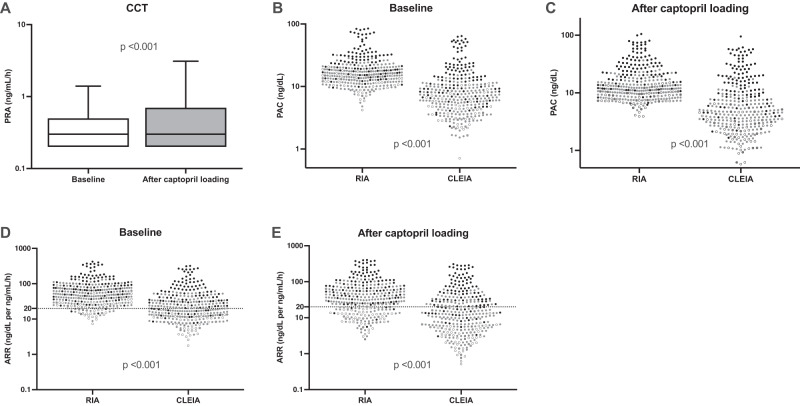
Changes of renin, aldosterone, and its ratio during the CCT. Changes in the plasma renin activity (PRA), plasma aldosterone concentration (PAC), and its ratio (ARR) during the captopril challenge test (CCT). Evaluation of these parameters were performed at baseline and 90 min after 50 mg captopril loading. PACs were measured using the novel chemiluminescent enzyme immunoassay (CLEIA) and radioimmunoassay (RIA, the conventional assay). **A** PRA changes during the CCT. **B** PACs at baseline. **C** PACs after captopril loading. **D** ARRs at baseline. **E** ARRs after captopril loading. In (**B**–**E**) panel, the symbols used show the participants as follows: black circle, unilateral primary aldosteronism (PA); grey circle, bilateral PA; open circle, essential hypertension; and open circle with a cross, PA patients without laterality identification. The *p*-values of all comparison using Mann Whitney U test were calculated as < 0.001 (**A**–**E**)

The RIA-PACs, PRAs, and RIA-ARRs at baseline of CCT (median with interquartile range) were 15.9 [12.0, 21.3] ng/dL, 0.3 [0.2, 0.5] ng/mL/h and 49.5 [29.3, 83.5] ng/dL per ng/mL/h, respectively (Fig. 2A–C). CLEIA-PACs measured in the same samples was 7.1 [4.5, 11.1] ng/dL and the calculated ARRs was 20.7 [12.0, 40.3] ng/dL per ng/mL/h, which were significantly lower than the RIA-PACs and its ARRs (*p* < 0.001 for both). After 50 mg captopril loading, RIA-PACs and CLEIA-PACs significantly decreased to 12.2 [9.1, 20.4] and 4.7 [2.8, 9.7] ng/dL, respectively (*p* < 0.001 for both). However, the PRAs increased to 0.3 [0.2, 0.7] ng/mL/h (*p* < 0.001; Fig. 2C, D). Accordingly, the RIA-ARRs and the CLEIA-ARRs decreased to 35.4 [17.1, 69.1] and 13.0 [5.3, 32.4] ng/dL per ng/mL/h, respectively (*p* < 0.001 for both; Fig. 2E). Among 238 patients with PA who demonstrated a positive CCT result in RIA (ARR > 20 ng/dL per ng/mL/h), 130 (54.6%) persistently presented with CLEIA-ARRs > 20 ng/dL per ng/mL/h after the captopril challenge. Contrastingly, other patients turned into “negative” in the CLEIA-based CCT; 67 (28.2%) had CLEIA-ARRs ranging between 10 and 20 ng/dL per ng/mL/h, and 41 (17.2%) had CLEIA-ARRs < 10 ng/dL per ng/mL/h. Of note, 22 (20.2%) patients with UPA were included in the “controversial” PA cases between RIA-based and CLEIA-based CCTs (Fig. 2E). The lowest CLEIA-based ARR at CCT was 3.32 ng/dL per ng/mL/h among patients with UPA. Of 95 UPA cases where resected adrenal specimens were available, 85 had APAs, and 10 had an aldosterone-producing nodule or multiple aldosterone-producing micronodules. The CLEIA-ARRs were higher in the APA cases than the other cases (49.1 [22.4, 123.8] vs. 24.2 [15.8, 50.1] ng/dL per ng/mL/h, respectively; *p* = 0.04). In addition, CLEIA-ARRs were correlated with the maximum diameter of APAs in the APA cases (Spearman’s *r* = 0.4426, *p* < 0.0001; Supplemental Fig. 1).

#### Assessment of the diagnostic ability of CLEIA-based CCT

**Fig. 3:**
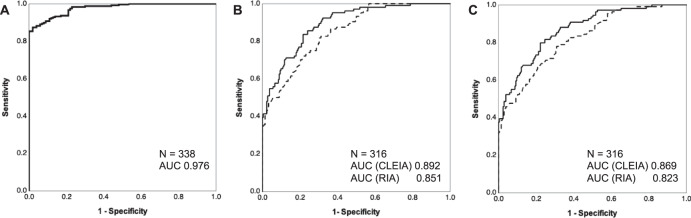
ROC curves of the CLEIA- and RIA-based CCT. The receiver operating characteristic (ROC) curve representing the discriminatory capacity of captopril challenge test (CCT) for primary aldosteronism (PA) in the novel chemiluminescent enzyme immunoassay (CLEIA) and radioimmunoassay (RIA, the conventional assay). The ROC curves and the areas under its curve (AUC) were evaluated in all the 338 cases in panel **A** and 316 cases after exclusion of PA patients without laterality identification in (**B**, **C**). **A** ROC curve of CLEIA-based CCT, targeting cases with a positive result of RIA-based CCT. **B** ROC curves of CLEIA-based and RIA-based CCTs for case detection of unilateral PA with positive RIA-based CCT. **C** ROC curves of CLEIA-based and RIA-based CCTs for case detection of all unilateral PA. In (**B**, **C**), the solid and dashed lines represent CLEIA-based CCT and RIA-based CCT, respectively

Next, we evaluated the diagnostic capacity of CLEIA-based CCT using ROC curve analysis, and compared it with RIA-based CCT. The AUC of CLEIA-based CCT of patients with positive CCT result (RIA-ARR > 20 ng/dL per ng/mL/h) was 0.976 with confidence interval ranging between 0.964 and 0.989 (Fig. 3A). The new cut-off value for CLEIA-based CCT was determined as an ARR of 8.2 ng/dL per ng/mL/h on the basis of the Youden index. The CLEIA-based ARR cut-off of 8.2 ng/dL per ng/mL/h demonstrated that the sensitivity and specificity were 0.874 and 0.980, respectively, for patients with PA who had positive RIA-based CCT results. Conversely, the CLEIA-based ARR cut-off of 20 ng/dL per ng/mL/h, the conventional cut-off value, had an equivalent diagnostic ability as the RIA-based ARR cut-off of 45.2 in CCT. In the context of detection in patients with UPA who had a positive RIA-based CCT result (RIA-ARR > 20 ng/dL per ng/mL/h), the CLEIA-based CCT had a larger AUC than the RIA-based CCT (0.892 vs. 0.851, *p* < 0.001; Fig. 3B and Supplemental Fig. 2). The optimal cut-off value, the CLEIA-ARR of 18.6 ng/dL per ng/mL/h, yielded sensitivity of 0.837 and specificity of 0.783 for patients with UPA who could be treated surgically. Inclusion of patients with UPA who were only positive for SIT did not affect the tendency of AUCs between CLEIA-based and RIA-based CCTs (0.869 vs. 0.823, *p* < 0.001; Fig. 3C, Supplementary Fig. 2).

## Discussion

Our findings clearly demonstrated that the measurement transition from RIA to new CLEIA impacted the diagnostic ability of the confirmatory test. Application of the conventional cut-off value could result in missing approximately half of the patients with PA in this CLEIA era. We propose an ARR of 8.2 ng/dL per ng/mL/h as an alternative cut-off value for CLEIA-based CCT to diagnose PA, which is consistent with the former criteria.

The establishment of LC-MS/MS measurement as the gold standard lead to the reassessment of plasma and urinary aldosterone levels in the clinical practice [21, 22]. Accumulating evidence has divulged that most of the commercial assays demonstrate higher aldosterone values compared to the actual values measured using LC-MS/MS [14, 15, 23, 24]. Studies from Australia and Japan demonstrated that the percentages of difference of median PACs between different RIAs and LC-MS/MS were 28.0% and 59.5%, respectively [14, 23]. The discrepancy in PACs between LC-MS/MS and other assays depends not only on the type of measurement assays used, but also on the concentration ranges of plasma aldosterone. A prospective study that compared PACs measured using enzyme-linked immunosorbent assay and LC-MS/MS showed that the range-dependent median differences in PACs were as follows: 65.8%, 51.4%, 13.3%, and 34.9% for < 10 ng/dL, between 10 and 20 ng/dL and 20–30 ng/dL, and > 30 ng/dL, respectively [24]. Few studies have also reported approximately 90% differences between PACs measured using RIA and LC-MS/MS [15]. These facts indicate that improvement in commercial aldosterone measurement and adjustment of cut-off values for each condition are required to precisely diagnose PA [25, 26].

Several researchers have raised concern regarding the immunoassay inaccuracy in aldosterone measurement [27]. The recently developed CLEIA methods for aldosterone measurement are easily available and reliable tool for PA practice. As forementioned, few studies with relatively small sample size (less than 100 samples) demonstrated that the measurement accuracy of the novel CLEIAs were consistent with that of LC-MS/MS [14–16]. The present study endorsed the fact that CLEIA-measured PACs strongly correlated with LC-MS/MS-measured PACs in a larger sample size as compared with the previous studies. The regression coefficient was 1.009, and the intercept and mean percentage of the difference were negligibly small in clinical settings. Our results, therefore, suggest that the novel CLEIA measurement for aldosterone can be considered as an alternative to LC-MS/MS. In addition, the CLEIA method employed in this study used the refined reagent for Accuraseed, an automated immunoassay system, which could measure the PAC and renin concentration simultaneously in just 10 min and 20 s [28]. The advantages of this CLEIA method include rapid results and onsite availability of the same parameters at reduced cost in comparison to LC-MS/MS. Therefore, we finally reached the starting line to understand the “real” pathogenesis related to RAAS in patients with high blood pressure.

As the first step, we developed the new ARR cut-off value of CCT for PA confirmation based on CLEIA. CCT is considered a suitable test to confirm renin-independent aldosterone production in hypertensive patients with low renin status. The cut-off value of CCT for PA has been defined as an ARR > 20 ng/dL per ng/mL/h, using the conventional RIA for aldosterone [18]. After indication of the novel CLEIA for aldosterone, the Japan Endocrine Society published a new guideline for PA, which sets a new CCT criterion of “provisional positive” for PA (ARR between 10 and 20 ng/dL per ng/mL/h) in addition to a “positive” category (ARR > 20 ng/dL per ng/mL/h) [13]. The “provisional positive” cut-off was designed based on the conversion formula from RIA-PAC to CLEIA-PAC, but not on the validation using actual blood samples. To the best of our knowledge, this is the first study to verify these cut-off values of CCT. Of note, we found that 28.2% and 17.2% of patients with positive RIA-based CCT fell into “provisional positive” and “negative” criteria of the CCT-based novel CLEIA, respectively. Furthermore, these groups harbored 17 and 5 UPA cases, where an adrenalectomy could lead to remission of PA. To appropriately provide examination and treatment for patients with PA in the current clinical situation, we proposed a new CLEIA-based ARR cut-off value of 8.2 ng/dL per ng/mL/h as a positive criterion of the CCT. This criterion is simple and would aid in detection of PA cases more compatible with the conventional diagnostic procedure than the current provisional criteria. These findings also imply that we must review previous cases where PA was excluded due to CLEIA-ARR at CCT < 10 ng/dL per ng/mL/h. Besides, our study revealed that CLEIA-ARR could more efficiently identify surgically-treatable PA cases than RIA-ARR. Further investigation to refine the CCT criteria for PA subtyping is required.

This study had a few limitations which need to be considered. First, the validation of novel CLEIA was mainly performed using baseline plasma samples and those obtained during CCT. Therefore, the measurement accuracy of the assay during SIT or AVS still remains unclear. Moreover, the CLEIA-ARR cut-off we proposed was developed for a criterion at 90 min after captopril loading. Cut-off values for 60 and 120 min after captopril challenge are to be determined in the future. Finally, we did not employ few confirmatory tests, including an upright furosemide test and an oral salt loading test, which may have influenced our recognition of PA and calculation of the assays’ diagnostic ability.

In conclusion, we successfully established a new cut-off value of CCT for PA confirmation based on the validation of the novel CLEIA by comparing it with the values obtained using LC-MS/MS and the conventional RIA. These findings will propel us forward into a New Age of PA management.

### Supplementary information

Supplemental material
