# Supplementary material for: Investigating the cut-off values of captopril challenge test for primary aldosteronism using the novel chemiluminescent enzyme immunoassay method: a retrospective cohort study
Source: Hypertens Res. 2024 Mar 8;47(5):1362–71. doi: 10.1038/s41440-024-01594-x (PMC11073978; doi:10.1038/s41440-024-01594-x)
Supplement: Supplementary file 1 — Supplemental material [file 41440_2024_1594_MOESM1_ESM.docx]

**Supplemental material**

**Investigating the Cut-off Values of Captopril Challenge Test for Primary Aldosteronism using the Novel Chemiluminescent Enzyme Immunoassay Method: A Retrospective Cohort Study**

Yuta Tezuka; Kei Omata; Yoshikiyo Ono; Kengo Kambara; Hiroki Kamada; Sota Oguro;

Yuto Yamazaki; Celso E. Gomez-Sanchez; Akihiro Ito; Hironobu Sasano; Kei Takase;

Tetsuhiro Tanaka; Hideki Katagiri; Fumitoshi Satoh.

**Supplemental figure 1:** The correlation between the maximal diameter of aldosterone-producing adenomas and the aldosterone-to-renin ratio after captopril loading.

In 85 cases with an aldosterone-producing adenoma (APA), the aldosterone-to-renin ratio after 50 mg captopril loading (CCT-ARR) and the maximal diameter (MD) of APAs were positively correlated by the Spearman’s rank analysis (Spearman’s r =0.4426, p<0.0001). The CCT-ARR was calculated based on plasma aldosterone concentration measured by the novel chemiluminescent enzyme immunoassay.

**Supplemental figure 2:** Schemas for study participants and ROC analysis.

A.

B.

(A) Overview of 338 study participants for investigation of the new cut-off value for captopril challenge test (CCT). Finally, 299 and 39 participants with low renin and high plasma aldosterone concentrations (PACs) were included as primary aldosteronism (PA) and essential hypertension (EH), respectively. The diagnosis of PA was established with CCT and/or saline infusion test (SIT). Most PA patients underwent adrenal venous sampling (AVS) to determine the laterality (unilateral or bilateral; UPA or BPA), while 22 avoided AVS due to personal reasons or their mild symptoms of PA.

(B) The target for comparative receiver operating characteristic (ROC) analysis of CCT-based laterality identification. We compared the diagnostic abilities of chemiluminescent enzyme immunoassay (CLEIA)-based CCT with its radioimmunoassay (RIA)-based one after excluding 22 PA patients who didn’t undergo AVS. First target was set as RIA-based CCT-positive UPA patients among 316 participants (the ROC analysis was shown in Figure 3B), while second target was defined as all UPA patients (the ROC analysis was shown in Figure 3C).
